# Nutritional and Lifestyle Behaviors Reported Following One Anastomosis Gastric Bypass Based on a Multicenter Study

**DOI:** 10.3390/nu15061515

**Published:** 2023-03-21

**Authors:** Shiri Sherf-Dagan, Reut Biton, Rui Ribeiro, Yafit Kessler, Asnat Raziel, Carina Rossoni, Hasan Kais, Rossela Bragança, Zélia Santos, David Goitein, Octávio Viveiros, Yitka Graham, Kamal Mahawar, Nasser Sakran, Tair Ben-Porat

**Affiliations:** 1 Department of Nutrition Sciences, School of Health Sciences, Ariel University, Ariel 40700, Israel; 2 Department of Nutrition, Assuta Medical Center, Tel Aviv 6971028, Israel; 3 Sackler Faculty of Medicine, Tel-Aviv University, Tel Aviv 6997801, Israel; 4 Multidisciplinary Center for Obesity Treatment, Hospital Lusíadas Amadora, 2724-002 Amadora, Portugal; 5 General Surgery Department Coordinator, Hospital Lusíadas Amadora, 2724-002 Amadora, Portugal; 6 Assia Medical Group, Assuta Medical Center, Tel Aviv 6971028, Israel; 7 Institute of Environmental Health, Faculty Medicine, University of Lisbon, 1649-028 Lisbon, Portugal; 8 Division of Surgery, Shamir Medical Center, Zerifin 70300, Israel; 9 H&TRC—Health & Technology Research Center, (ESTeSL) Escola Superior de Tecnologia da Saúde de Lisboa, 1990-096 Lisbon, Portugal; 10 Department of Surgery C, Sheba Medical Center, Tel Hashomer, Ramat Gan 5266202, Israel; 11 Faculty of Health Sciences and Wellbeing, University of Sunderland, Sunderland SR1 3SD, UK; 12 Bariatric Surgical Unit, Sunderland Royal Hospital, Sunderland SR4 7TP, UK; 13 Department of Surgery, Holy Family Hospital, Nazareth 1600100, Israel; 14 The Azrieli Faculty of Medicine Safed, Bar-Ilan University, Ramat Gan 5290002, Israel; 15 Department of Health, Kinesiology, and Applied Physiology, Concordia University, Montréal, QC H4B 1R6, Canada; 16 Montreal Behavioural Medicine Centre (MBMC), Centre Intégré Universitaire de Santé et de Services Sociaux du Nord-de-l’Île-de-Montréal (CIUSSS-NIM), Montréal, QC H4J 1C5, Canada

**Keywords:** one anastomosis gastric bypass, nutrition, lifestyle behaviors, adherence

## Abstract

This study aimed to describe nutritional and lifestyle parameters following one-anastomosis gastric bypass (OAGB). A multicenter study among OAGB patients across Israel (*n* = 277) and Portugal (*n* = 111) was performed. Patients were approached according to the time elapsed since surgery. An online survey with information regarding demographics, anthropometrics, and nutritional and lifestyle aspects was administered in both countries simultaneously. Respondents from Israel (pre-surgery age of 41.6 ± 11.0 years, 75.8% females) and Portugal (pre-surgery age of 45.6 ± 12.3 years, 79.3% females) reported changes in their appetite (≤94.0% and ≤94.6%), changes in their taste (≤51.0 and ≤51.4%), and intolerance to specific foods (i.e., red meat, pasta, bread, and rice). Bariatric surgery-related eating recommendations were generally followed well, but a trend toward lower adherence was evident in groups with longer time elapsed since surgery in both countries. Most respondents from Israel and Portugal reported participation in follow-up meetings with a surgeon (≤94.0% and 100%) and a dietitian (≤92.6% and ≤100%), while far fewer reported participation in any follow-up meeting with a psychologist/social worker (≤37.9% and ≤56.1%). Patients following OAGB might experience changes in appetite, taste, and intolerance to specific foods. Adherence to bariatric surgery-related eating recommendations is not always satisfying, especially in the longer term post-surgery.

## 1. Introduction

Bariatric surgery (BS) is a validated treatment modality for severe obesity that is usually considered when other modalities to lose weight have failed [1]. BS includes several procedure types with respective pros and cons [2]. One-anastomosis gastric bypass (OAGB) surgery is a bariatric procedure that is gaining popularity worldwide, but mainly in specific regions [3]. It is considered a “combined procedure” which includes both “restrictive” and “malabsorptive” components [4]. Presently, there are considerable variabilities in surgical technique administration, with the biliopancreatic limb length being one of the main debates among surgeons performing this procedure [2]. Nevertheless, several controversies as to the results and implications of this procedure exist, especially regarding the longer term [5]. One issue for which information is currently lacking are the nutritional implications of OAGB [4]. Although an increasing number of studies present information on nutritional status in terms of nutritional deficiencies and malnutrition following OAGB [2,6,7,8,9,10,11,12,13,14,15], data on other nutritional-related topics that could impact patients’ adjustments including aspects that could impact food intake and compliance with the nutritional and lifestyle recommendations are scarce [9,16,17]. As changes in eating behaviors and lifestyle habits are essential for optimal bariatric procedure outcomes, collecting data on this information is crucial [18]. Therefore, we aimed to gain information on nutritional and lifestyle parameters from two samples of OAGB patients living in different countries. 

## 2. Materials and Methods

A multicenter study was performed in Israel and Portugal. OAGB patients were approached by study teams in each center according to a patient list based on time elapsed since the surgery (i.e., 1–6 months (1–6 M), 6–12 months (6–12 M), and 1–5 years (1–5 Y) post-surgery). Recruitment of patients according to defined time frames since the surgery was performed due to a priori expected differences in clinical and behavioral outcomes in different time periods since surgery. Inclusion criteria included age of ≥18 years and primary OAGB in the last 5 years, and exclusion criteria included revisional BS, present pregnancy, and lack of capacity to consent. All eligible patients were informed about the study, asked to consider participating, and informed that participation was voluntary. Patients who gave their verbal consent to participate in the study were asked to complete an anonymous online survey which was delivered using SurveyMonkey^®^ software through email/SMS message. An invitation to participate in the study was re-sent to non-responders after 4 weeks. Data were collected between 26 June 2020 and 9 May 2021. A research coordinator led the local study management in each country. Ethics approval by local institutional review boards of each medical center was received. The work was reported based on the STROCSS criteria [19]. 

### 2.1. Survey-Included Data

The survey included data on demographics, medical condition, anthropometrics (i.e., weight history and self-reported weight and height, followed by a calculation of body mass index (BMI), and excess weight loss (EWL) percentages [20]), and nutritional, lifestyle, and gastrointestinal parameters. The English version of the survey is presented in Supplementary Materials. The survey was distributed in Hebrew (for the study in Israel) and Portuguese (for the study in Portugal). Linguistic translation and cultural adaptation of items were performed according to recommended methodologies when needed [21,22]. This paper reports specifically on nutritional- and lifestyle-related sections within the original full-length questionnaire, as detailed below. Due to differences in OAGB technique between the countries, outcomes are presented by country. 

### 2.2. Food Tolerance

**Figure 1:**
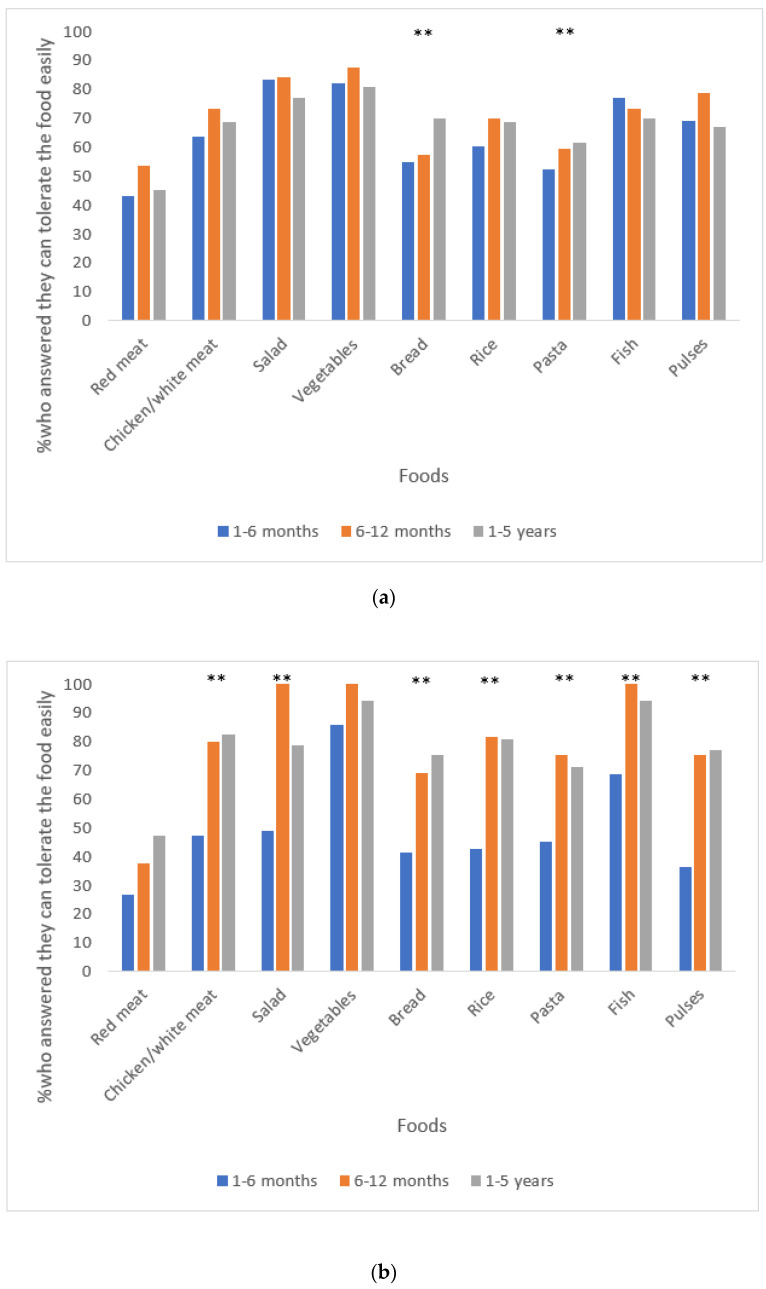
(**a**): Tolerance to different food items by time elapsed since surgery (Israel) ^1,2,3^. (**b**): Tolerance to different food items by time elapsed since surgery (Portugal) ^1,2,3^. ^1^ Data were available for participants from Israel and Portugal for *n* = 101 and *n* = 36 respondents between 1–6 months post-surgery, *n* = 56 and *n* = 16 respondents between 6–12 months post-surgery, and *n* = 104 and *n* = 52 respondents between 1–5 years post-surgery. ^2^ Patients were asked how they can eat each of the following food items (easily/with some difficulties/not at all). ^3^ Pulses (e.g., lentils, chickpeas, peas, beans) were added to the original questionnaire for quick assessment of food tolerance after bariatric surgery. ** Significant differences between groups divided by time elapsed since surgery.

Food tolerance was assessed using a validated questionnaire for quick assessment of food tolerance after BS [9,23] with minor modifications (i.e., pulses were added to the list of food items, but not included in the final scoring) (Figure 1a,b). Cumulatively, scores range from 1 (lowest score) to 27 (highest score) [23]. Furthermore, patients were also asked if they have other food restrictions due to health, cultural, religious, ethical, and/or belief reasons. The questionnaire for quick assessment of food tolerance after BS was previously translated to Hebrew and Portuguese and has been used in numerous studies in both languages [9,24,25,26,27,28].

### 2.3. Appetite, Taste, Smell, and Food Aversion Alternations after OAGB 

Appetite, taste, smell, and food aversion alternations after OAGB were assessed using selected items obtained from previously published validated questionnaires on these topics [29,30,31]. 

### 2.4. Compliance with the BS Eating Recommendations

Compliance with the BS eating recommendations was assessed by asking the patients to what extent they were following the BS eating recommendations during the last month (no/partially/always) [32].

### 2.5. Compliance with the BS Lifestyle Recommendations 

Compliance with the BS lifestyle recommendations was assessed using questions targeting smoking habits, physical activity, and frequency of multivitamin intake. Additionally, patients were asked regarding participation in a follow-up regime with the multidisciplinary team and/or support groups since the surgery. Patients were also asked if they took part in a local BS group through social media and regarding the reasons for participation in these groups.

### 2.6. Statistical Analyses

Statistical analyses were performed using SPSS software (version 26). Tests for normality distribution were used for continuous variables. Continuous variables are presented as means ± SD or median with interquartile range as needed, and categorical variables as proportions. To test differences in continuous variables between the three time-points post-surgery one-way ANOVA or the Kruskal–Wallis test were used when needed. For comparison of dichotomous or categorical variables between the three time-points post-surgery the chi-square test or Fisher’s exact test were performed. The level of significance for all analyses was set at *p* < 0.05 and Bonferroni correction was applied when needed.

Power calculation. When applying a sample size of *n* = 277 (Israel) or *n* = 111 (Portugal), a 0.05 two-sided alpha level, and a medium effect size (Cohen d = 0.5) [33] in G*power software (version 3.1.9.4) for one sample *t*-tests, a power of >0.999 was calculated. 

## 3. Results

### 3.1. Study Participant Characteristics 

A total of *n* = 277 responses from Israel (*n* = 109, *n* = 59, and *n* = 109 for 1–6 M, 6–12 M, and 1–5 Y groups, respectively) and *n* = 111 responses from Portugal (*n* = 40, *n* = 17, and *n* = 54 for 1–6 M, 6–12 M, and 1–5 Y groups, respectively) were obtained. Mean months elapsed since surgery for responses from Israel and Portugal were 3.2 ± 1.7 and 2.5 ± 1.7 (1–6 M group), 9.2 ± 1.6 and 8.6 ± 1.5 (6–12 M group), and 33.0 ± 15.1 and 27.3 ± 13.0 (1–5 Y groups).

Mean reported pre-surgery age, BMI, and gender distribution (% female) were 41.6 ± 11.0 and 45.6 ± 12.3 years, 41.2 ± 4.8 and 40.1 ± 5.6 kg/m^2^, and 75.8 and 79.3% for respondents from Israel and Portugal, respectively. Pre-surgery characteristics of the survey respondents grouped by time elapsed since surgery and by country are presented in Table 1. Respondents from Israel and Portugal reported pre-surgery prevalence of 17.0% and 19.3% type 2 diabetes, 25.6% and 44.0% hypertension, and 39.0% and 51.4% dyslipidemia.

### 3.2. Anthropometrics

Respondents from Israel and Portugal presented mean %EWL of 51.0 ± 19.9 and 62.4 ± 26.5% (1–6 M group), 89.0 ± 22.0 and 86.2 ± 21.4% (6–12 M group), and 89.9 ± 23.6 and 98.2 ± 20.9% (1–5 Y group), respectively (*p* < 0.001 for both countries). The median of weight-regain at 1–5 Y, calculated as current weight minus nadir weight, was 2.8 kg (range: 0–35.0 kg) and 2.0 kg (range: 0–23.0 kg) among respondents from Israel and Portugal, respectively (Table 1). 

### 3.3. Food Tolerance, Appetite, Taste, Smell, and Food Aversion Alternations 

Tolerance to specific food items grouped by time elapsed since surgery per country is presented in Figure 1a,b. Aspects that could impact food intake grouped by time elapsed since surgery and by country are presented in Table 2. The great majority of respondents from Israel and Portugal in all time-elapsed-since-surgery groups reported changes in their appetite (≤94.0% and ≤94.6%), while only a minority reported eating less due to bad taste or smell (≤23.5% and ≤9.8%). Experiencing changes in taste was reported by 51.0% and 51.4% (1–6 M group), 46.6% and 43.8% (6–12 M group), and 51.0% and 38.0% (1–5 Y group) of respondents from Israel and Portugal, respectively (Table 2).

### 3.4. Compliance with the BS Nutritional and Lifestyle Recommendations

**Figure 2:**
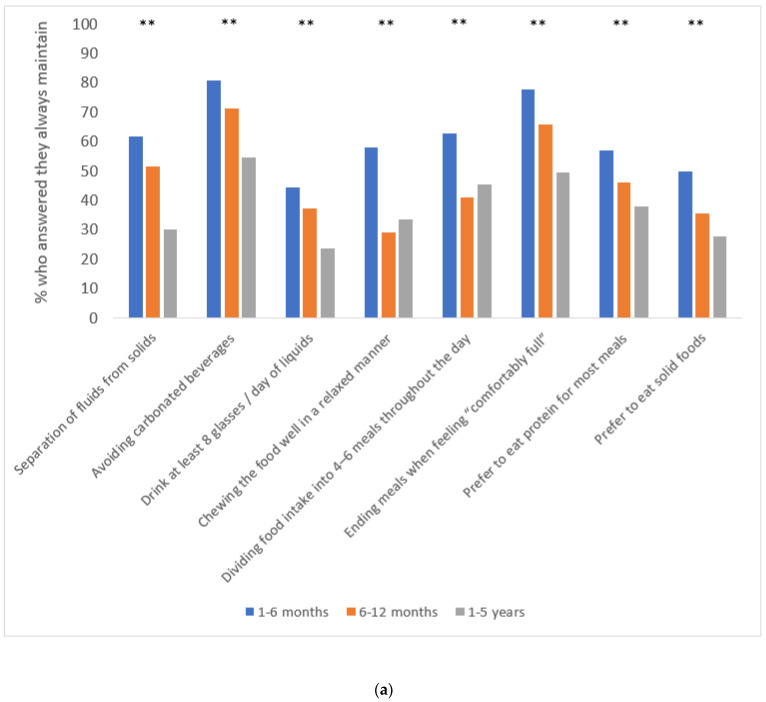

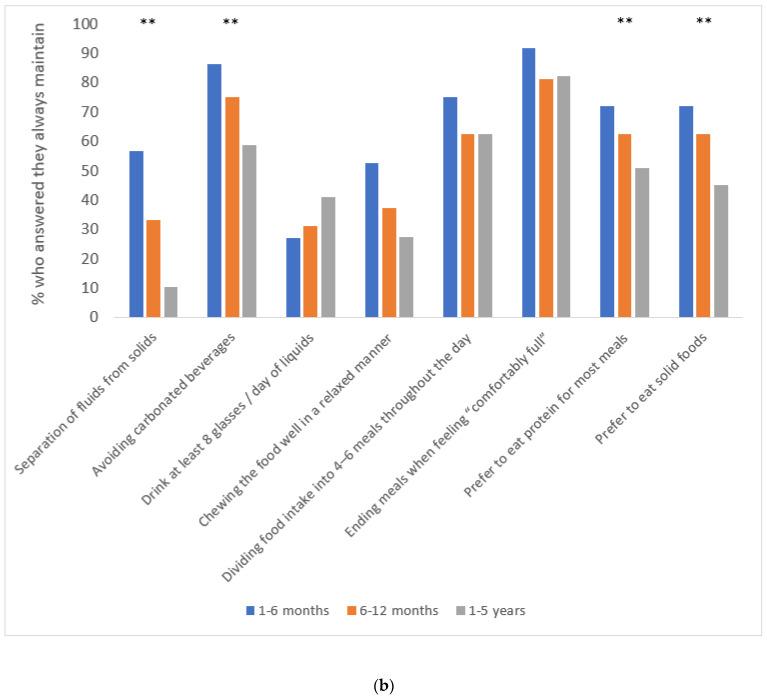
(**a**): Adherence to bariatric surgery eating recommendations grouped by time elapsed since surgery (Israel) ^1,2,3,4^. (**b**): Adherence to bariatric surgery eating recommendations grouped by time elapsed since surgery (Portugal) ^1,2,3,4^. ^1^ Data were available for participants from Israel and Portugal for *n* = 100 and *n* = 37 respondents between 1–6 months post-surgery, *n* = 56 and *n* = 16 respondents between 6–12 months post-surgery, and *n* = 103 and *n* = 51 respondents between 1–5 years post-surgery, respectively. ^2^ Patients were asked if they keep the following recommended behaviors for the last month (always maintained/partially maintained/not maintained). ^3^ A glass of drink was considered as 200 mL. ^4^ Preference to eat solid food items (e.g., boiled egg, chicken breast, salad) over soft or crunchy food items (e.g., ice cream, cookies, cakes, cookies) in most meals. ** Significant differences between time elapsed since surgery groups.

The majority of respondents from Israel and Portugal reported adherence to most of the BS eating recommendations within the 1–6 M group. However, a trend toward lower adherence to most of them was noticed within groups with longer time elapsed since surgery in each country (Figure 2a,b). Postoperative health behaviors grouped by time elapsed since surgery and by country are presented in Table 3. Daily intake of multivitamin was reported to be lower within groups with longer time elapsed since surgery among respondents from Israel (90.0%, 84.9%, and 63.0%, within 1–6 M, 6–12 M, and 1–5 Y groups, respectively, *p* < 0.001), but similar within groups with different time elapsed since surgery among respondents from Portugal (90.5%, 90.0%, and 90.9%, within 1–6 M, 6–12 M, and 1–5 Y groups, respectively, *p* = 1.000). Attendance to the follow-up regime grouped by time elapsed since surgery and by country is presented in Table 4. The majority of respondents from Israel and Portugal in all time-elapsed-since-surgery groups reported participation in follow-up meetings with a surgeon (≤94.0% and 100%) and a dietitian (≤92.6% and ≤100%), while far fewer reported participation in any follow-up meeting with a psychologist/social worker (≤37.9% and ≤56.1%) (Table 4).

## 4. Discussion

In the present study, we aimed to gain information on nutritional and lifestyle parameters following OAGB including food tolerance, eating difficulties, taste and smell changes, and adherence to the BS nutritional and lifestyle recommendations. In terms of anthropometric results, satisfying weight outcomes were reported within all the time-elapsed-since-surgery groups in each country. Nonetheless, the median weight regain reported by respondents from both countries at the mid-term post-surgery was 2–3 kg, which is within the acceptable range up to 5 years following bariatric procedures [34]. However, the range of reported weight-regain was wide between individuals in both countries. 

Adherence to the BS eating recommendations, adequate follow-up support, and physical activity were all found to be associated with surgical outcomes [18,35,36]. Therefore, collecting data on these parameters following surgery is important to identify patients prone to poorer outcomes that may be in need of behavioral and nutritional intervention. In the present study, relatively high adherence to the BS eating recommendations was reported within the 1–6 M group, but a trend toward lower adherence to most of them was noticed within groups with longer time elapsed since surgery in each country. This result is in accordance with previous studies which found a similar trend [18,37]. One plausible explanation for this phenomenon could be “behavioral fatigue”, as multiple health behavior changes are required in the long-term following bariatric procedures [18]. In addition, patients report that during the “honeymoon period” when weight loss is drastic and rapid with the “surgery doing the work” in limiting appetite, portion sizes, and interest in foods, it is easier to follow the BS rules compared to later periods when weight has stabilized and “the work begins” as physiological adjustments occur resulting in increased hunger, portion sizes, and interest in foods [18,38]. Food intolerances may impact the diet quality of patients who undergo different types of bariatric procedures, but some adjustments and adaptations of the gastrointestinal system probably occur over time [16,39]. A recently published systematic review found that red meat, rice, bread, pasta, dairy, and fibrous vegetables were the most prevalent reported food intolerances following different types of bariatric procedures [39]. In the present study, we observed food intolerance mainly for red meat, pasta, bread, and rice, but for most a trend toward better tolerance was noticed within groups with a longer time passed since surgery in both countries. Additionally, in both countries the mean food tolerance score was found to be higher within groups with longer time elapsed since surgery in comparison to within the 1–6 M group. Nonetheless, it seems that the prevalence of food intolerance for specific food items was different between respondents from both countries which might reflect differences in eating habits and food preferences. 

Further nutritional aspects that should be taken into consideration following bariatric procedures are changes in appetite, taste, and smell. In the present study, changes in appetite were notable among groups with different elapsed times since surgery in each country. Gut hormones, which affect appetite and satiety, may play a causal role in mediating weight loss following BS, but the extent of their role following OAGB is presently less clear [40,41]. Experiencing changes in taste was reported by about half of the respondents in each country, while changes in the taste of water, coffee, and sweets were the most prevalent reported in free text. Experiencing changes in smell was reported by a minority of respondents in each country. Changes in taste and smell perceptions are probably related to adipose–gut–brain-axis modifications which occur following BS and may influence food preferences [42,43]. However, the extent of sensory changes presumably varies between different patients and bariatric procedures. A previous cross-sectional study among 103 Roux-en-Y gastric bypass (RYGB) patients with a median follow-up of 19 months post-surgery, which used similar tools to assess such outcomes, revealed higher postoperative sensory changes in appetite, taste, and smell [29]. Nevertheless, another cross-sectional study, which included 126 patients who underwent RYGB or sleeve gastrectomy (SG) with a mean of 5.0 ± 4.0 years since surgery and used similar tools to assess such outcomes, revealed similar trends [42]. Collectively, future studies should further investigate changes in appetite, taste, and smell following OAGB, preferably using validated and objective methodologies [44], as these may significantly impact the dietary patterns and nutritional outcomes of patients.

As the irregular intake of supplementation may trigger nutritional deficiencies following bariatric procedures and impose health risks, lifelong supplementation is required [45,46]. In the present study, we focused on multivitamin as a marker of adherence to the ”supplementation regime”, although protocols, products, and dosages might be diverse between locations. Daily intake of a multivitamin was reported to be lower within groups with longer time elapsed since surgery among respondents from Israel, but similar within groups with different times elapsed since surgery among respondents from Portugal. This result might reflect differences in patient education, health policies, and cost issues. Nonetheless, our results are more positive than a previous study among 128 OAGB patients which found that during three years of follow-up ≥59% reported compliance to the “supplementation regime”, defined as 5 intakes/week or more [17]. Moreover, adherence to taking supplements following bariatric procedures was previously found to decrease over time [39,46,47,48]; plausible explanations could be patient-related, product-related, economics-related, and healthcare-related. According to a recently published multicenter survey study which was based on the patients’ perspective, factors affecting adherence to multivitamin intake after surgery include mainly forgetfulness, gastrointestinal side effects, unpleasant taste, smell and/or size of the pill, and high costs. Therefore, these factors should be taken into consideration while educating and treating patients who undergo bariatric procedures [47]. 

In the present study, a minority of respondents from both countries reported reaching the physical activity target of at least 150 min/week of exercising, which is lower than reported in a previous study on 86 OAGB patients 12–20 months post-OAGB [10]. Barriers to perform physical activity among BS patients include both internal barriers (i.e., motivational and physical factors) and external barriers (i.e., resources, support, time, and weather) [18,49]. Reduction in sedentary activities while increasing performance of a physical activity is a known strategy to attenuate weight regain and promote general health [50,51]. Therefore, physical activity promotion and reducing barriers should be an important area of focus for clinicians [18].

The great majority of respondents reported meetings with a surgeon and a dietitian, while much fewer reported meetings with a psychologist/social worker in both countries. Nonetheless, it seems that utilization of BS groups through social media was far more popular among respondents from Israel. Follow-up visits are difficult to enforce post-operatively [18], thus identifying adherence barriers is crucial. The main identified causes of attrition from follow-up meetings after bariatric procedures are probably related to logistic issues, but also lack of awareness of their importance [52]. Therefore, along with patient education and engagement, efforts should be made to address these barriers, and digital communication methods should be utilized to diminish barriers such as distance, time, and cost [18,32,53].

The major strength of this study includes the use of acceptable tools to assess nutrition and lifestyle outcomes. Moreover, the inclusion of patients with three time intervals since the surgery is one of its strengths, as sensory changes and adherence to clinical recommendations could gradually decrease over time [18,37,42]. However, there are some limitations to be mentioned. First, reporting bias and more specifically social-desirability bias cannot be ruled out. Second, data were collected during the COVID-19 pandemic which was related to changes in eating and lifestyle behaviors by a great part of the world population [54]. Third, objective measurements were not collected, thus data on nutritional deficiencies or nutrient intake could not be assessed objectively. 

## 5. Conclusions 

Patients following OAGB might experience changes in appetite, taste, and intolerance to specific food items. Adherence to BS nutritional recommendations is not always satisfying, especially in the longer term after surgery. Although the trend for most aspects that could impact food intake outcomes in time-elapsed-since-surgery groups was alike between respondents from Israel and Portugal, some differences regarding adherence to specific BS eating and lifestyle recommendations, attendance to meetings with the multidisciplinary team, and utilization of BS groups through social media were noticed. These results might reflect differences in health policies, clinical practices, eating habits, and culture between participants from the two different countries. Future high-quality prospective long-term studies are needed to increase our knowledge regarding the effect of OAGB on a broad spectrum of nutritional and lifestyle outcomes. 

## Figures and Tables

**Table 1:** Self-reported demographic and anthropometric parameters grouped by time elapsed since surgery and by country.

Parameter ^1,2^		1–6 Months Post-Surgery	6–12 Months Post-Surgery	1–5 Years Post-Surgery	*p* Value
Pre-surgery self-reported demographic parameters					
Age (years)	Israel	40.2 ± 10.4	38.9 ± 11.1	44.4 ± 11.0	0.002 ^b,c^
	Portugal	45.5 ± 11.0	48.7 ± 10.0	44.7 ± 13.8	0.501
Gender (% women)	Israel	77.1	83.1	70.6	0.186
	Portugal	77.5	76.5	81.5	0.853
Marital status (% married)	Israel	67.9	71.2	65.1	0.722
	Portugal	55.0	52.9	42.6	0.457
Pre-surgery self-reported anthropometric parameters					
Weight (kg)	Israel	113.8 ± 17.9	113.1 ± 13.6	117.9 ± 23.0	0.220
	Portugal	105.8 ± 15.9	122.1 ± 23.8	112.6 ± 20.4	0.019 ^a^
Height (m)	Israel	1.67 ± 0.09	1.67 ± 0.08	1.68 ± 0.09	0.472
	Portugal	1.68 ± 0.08	1.68 ± 0.07	1.66 ± 0.09	0.401
BMI (kg/m^2^)	Israel	40.9 ± 4.3	40.9 ± 4.2	41.6 ± 5.6	0.526
	Portugal	37.5 ± 4.0	42.9 ± 6.9	40.9 ± 5.5	0.001 ^a,b^
Post-surgery self-reported anthropometric parameters					
Weight (kg)	Israel	91.9 ± 16.0	75.4 ± 11.1	77.3 ± 16.2	<0.001 ^a,b^
	Portugal	84.8 ± 13.3	80.5 ± 12.3	71.5 ± 13.2	<0.001 ^b,c^
BMI (kg/m^2^)	Israel	33.0 ± 4.2	27.3 ± 3.9	27.2 ± 4.3	<0.001 ^a,b^
	Portugal	30.1 ± 3.4	28.4 ± 3.8	26.0 ± 3.6	<0.001 ^b,c^
EWL (%)	Israel	51.0 ± 19.9	89.0 ± 22.0	89.9 ± 23.6	<0.001 ^a,b^
	Portugal	62.4 ± 26.5	86.2 ± 21.4	98.2 ± 20.9	<0.001 ^a,b^
Weight regain (kg) (median (interquartile range)) ^3^	Israel	-	-	2.8 (1.0–6.0)	-
	Portugal	-	-	2.0 (0.9–7.0)	-

Abbreviations: Body mass index (BMI), excess weight loss (EWL). ^1^ Values are expressed as mean ± SD, unless otherwise stated. ^2^ Data were available for participants from Israel and Portugal for *n* = 109 and *n* = 40 respondents between 1–6 months post-surgery, *n* = 59 and *n* = 17 respondents between 6–12 months post-surgery, and *n* = 109 and *n* = 54 respondents between 1–5 years post-surgery, respectively. ^3^ Calculated as the gap between current weight and weight nadir reported by respondents. ^a^ Significant differences between the groups 1–6 months and 6–12 months post-surgery. ^b^ Significant differences between the groups 1–6 months and 1–5 years post-surgery. ^c^ Significant differences between the groups 6–12 months and 1–5 years post-surgery.

**Table 2:** Aspects that could impact food intake grouped by time elapsed since surgery and by country.

Parameters ^1^		1–6 Months Post-Surgery	6–12 Months Post-Surgery	1–5 Years Post-Surgery	*p* Value
Aspects that could impact food intake ^2^					
Food Tolerance score	Israel	21.7 ± 3.6	23.1 ± 2.9	22.2 ± 3.5	0.062
	Portugal	19.0 ± 4.3	24.1 ± 2.2	23.5 ± 3.8	<0.001 ^a,b^
Food or drinks that are repulsive or intolerable (% responded yes) ^3^	Israel	47.0	39.3	51.0	0.370
	Portugal	32.4	37.5	52.9	0.140
Socio-cultural aspects that impact food intake (% responded yes)	Israel	31.7	32.1	33.7	0.953
	Portugal	32.4	20.0	28.8	0.669
Changes in appetite, taste, and smell ^2^					
Any change in appetite (% responded yes)	Israel	94.0	73.2	81.4	0.001 ^a,b^
	Portugal	94.6	93.8	90.4	0.879
Eating less food because of being less hungry (% responded yes)	Israel	81.0	76.8	73.5	0.449
	Portugal	94.6	93.8	90.2	0.879
Eating less because of bad taste or smell (% responded yes)	Israel	23.0	21.4	23.5	0.955
	Portugal	8.1	6.7	9.8	1.000
Any change in taste of food and drinks (% responded yes) ^4^	Israel	51.0	46.6	51.0	0.833
	Portugal	51.4	43.8	38.0	0.463
Any change in smell (% responded yes)	Israel	19.0	16.1	26.5	0.240
	Portugal	13.5	12.5	14.0	0.988

^1^ Values are expressed as mean ± SD, unless otherwise stated. ^2^ Data were available for participants from Israel and Portugal for *n* = 100 and *n* = 37 respondents between 1–6 months post-surgery, *n* = 56 and *n* = 16 respondents between 6–12 months post-surgery, and *n* = 102 and *n* = 51 respondents between 1–5 years post-surgery, respectively. ^3^ *n* = 107 and *n* = 39 participants from Israel and Portugal answered also in free text; of those, *n* = 19 and *n* = 0 reported an aversion to water, *n* = 17 and *n* = 6 reported an aversion to carbonated beverages, *n* = 11 and *n* = 9 reported an aversion to sweet drinks or food, *n* = 17 and *n* = 1 reported an aversion to coffee, *n* = 11 and *n* = 1 reported an aversion to milk, *n* = 8 and *n* = 2 reported an aversion to eggs or omelet, *n* = 3 and *n* = 5 reported an aversion to alcohol, while the rest gave other varied answers. ^4^ *n* = 94 and *n* = 39 participants from Israel and Portugal answered also in free text; of those *n* = 20 and *n* = 5 reported a change in sweet drinks or food, *n* = 22 and *n* = 0 reported a change in water taste, and *n* = 14 and *n* = 1 reported a change in coffee taste, while the rest gave other varied answers. ^a^ Significant differences between the groups 1–6 months and 6–12 months post-surgery. ^b^ Significant differences between the groups 1–6 months and 1–5 years post-surgery.

**Table 3:** Postoperative health behaviors grouped by time elapsed since surgery and by country.

Parameters		1–6 Months Post-Surgery	6–12 Months Post-Surgery	1–5 Years Post-Surgery	*p* Value
Smoking status ^1^					
Currently smoking (%)	Israel	13.8	16.9	20.2	0.630
	Portugal	12.8	0	5.6	0.353
Physical activity during the last month ^2^					
Reported exercising ≥150 min/week (%)	Israel	15.0	22.2	14.7	0.429
	Portugal	16.7	6.7	11.8	0.594
Supplementation usage during the last month ^3^					
Multivitamin (% reported daily usage)	Israel	90.0	84.9	63.0	Israel: <0.001 ^b,c^ Portugal: 1.000
	Portugal	90.5	90.0	90.9	
Multivitamin (% reported weekly usage)	Israel	2.0	0	6.0	
	Portugal	0	0	0	
Multivitamin (% reported monthly or no usage)	Israel	8.0	15.1	31.0	
	Portugal	9.5	10.0	9.1	

^1^ Data were available for participants from Israel and Portugal for *n* = 109 and *n* = 39 respondents between 1–6 months post-surgery, *n* = 59 and *n* = 17 respondents between 6–12 months post-surgery, and *n* = 109 and *n* = 54 respondents between 1–5 years post-surgery, respectively. ^2^ Data were available for participants from Israel and Portugal for *n* = 100 and *n* = 36 respondents between 1–6 months post-surgery, *n* = 54 and *n* = 15 respondents between 6–12 months post-surgery, and *n* = 102 and *n* = 51 respondents between 1–5 years post-surgery, respectively. ^3^ Data were available for participants from Israel and Portugal for *n* = 100 and *n* = 21 respondents between 1–6 months post-surgery, *n* = 53 and *n* = 10 respondents between 6–12 months post-surgery, and *n* = 100 and *n* = 33 respondents between 1–5 years post-surgery, respectively. ^b^ Significant differences between the groups 1–6 months and 1–5 years post-surgery. ^c^ Significant differences between the groups 6–12 months and 1–5 years post-surgery.

**Table 4:** Attendance to the follow-up regime grouped by time elapsed since surgery and by country.

Parameters		1–6 Months Post-Surgery	6–12 Months Post-Surgery	1–5 Years Post-Surgery	*p* Value
Participation in follow-up meetings with the bariatric team (%) ^1^					
Registered dietitian					
Any meeting (% responded yes)	Israel	89.0	92.6	85.7	0.436
	Portugal	100	100	93.9	0.278
Bariatric surgeon					
Any meeting (% responded yes)	Israel	94.0	83.3	81.8	0.026 ^b^
	Portugal	100	100	100	-
Psychologist/Social worker					
Any meeting (% responded yes)	Israel	22.7	31.5	37.9	0.072
	Portugal	36.7	26.7	56.1	0.086
Pharmacist					
Any meeting (% responded yes)	Israel	58.6	52.9	30.4	<0.001 ^b,c^
	Portugal	11.5	23.1	8.1	0.324
Attendance in support group meetings					
% who responded yes	Israel	4.0	7.4	21.0	0.001 ^b^
	Portugal	0	6.7	3.9	0.263
Participate in local/national bariatric surgery groups through social media					
% who responded yes	Israel	76.0	72.2	48.5	<0.001 ^b,c^
	Portugal	22.2	6.3	23.5	0.308

^1^ Data were available for participants from Israel and Portugal for *n* = 100 and *n* = 37 respondents between 1–6 months post-surgery, *n* = 54 and *n* = 16 respondents between 6–12 months post-surgery, and *n* = 100 and *n* = 51 respondents between 1–5 years post-surgery, respectively. ^b^ Significant differences between the groups 1–6 months and 1–5 years post-surgery. ^c^ Significant differences between the groups 6–12 months and 1–5 years post-surgery.

## Data Availability

Data are available on reasonable request. All data relevant to the study are included in the article or uploaded as Supplementary Information.

## Supplementary Materials

The following supporting information can be downloaded at: https://www.mdpi.com/article/10.3390/nu15061515/s1, File S1: Survey for One Anastomosis Gastric Bypass patients.
