# Supplementary material for: Nutritional and Lifestyle Behaviors Reported Following One Anastomosis Gastric Bypass Based on a Multicenter Study
Source: Nutrients. 2023 Mar 21;15(6):1515. doi: 10.3390/nu15061515 (PMC10053792; doi:10.3390/nu15061515)
Supplement: Supplementary file 1 [file nutrients-15-01515-s001.zip › nutrients-2205917-supplementary.pdf]

Survey for One Anastomosis Gastric Bypass patients

As part of a study on gastrointestinal and nutritional parameters among Mini Gastric Bypass/One Anastomosis Gastric Bypass patients, we would kindly ask you to fill out this questionnaire.
The questionnaire is completely anonymous and takes approximately 15 minutes to complete. Completing the questionnaire is not mandatory. At any time, you can stop participating for any reason by closing the survey link. In this case, the information entered will not be saved.

1. Demographics

- What was the date of the bariatric surgery? (month/day/year) __/__/____
- Time since the bariatric surgery (in months) __
- Date of birth: (month/day/year) __/__/____
- Your current age (in years) __
- Your gender: male/female
- What is your current marital status?
  - Single
  - Domestic partnership
  - Married
  - Separated
  - Divorced
  - Widowed
- What was your marital status before the bariatric surgery?
  - Single
  - Domestic partnership
  - Married
  - Separated
  - Divorced
  - Widowed
- How many children do you have? ___

- What is the highest degree or level of education you have completed?
  - Less than a high school diploma
  - High school degree or equivalent
  - Technical training with no degree
  - Bachelor's degree
  - Master's degree
  - Doctor of Medicine (MD)/Doctor of Veterinary (DVM)/Doctor of Philosophy (PhD)
  - Other (please state):_____________
- What is your current occupational status: Full time/Part time/Unemployed

2. Medical status

** Diagnosis of morbidity by a physician or taking medication for the morbidity.

- What is your smoking status: current smoker/past smoker/never smoked?
- Are you currently diagnosed with high blood pressure or hypertension**? Yes/No
- Are you currently diagnosed with high blood cholesterol **? Yes/No
- Are you currently diagnosed with type 2 diabetes **? Yes/No
- Were you diagnosed with high blood pressure or hypertension before the bariatric surgery**? Yes/No
- Were you diagnosed with high blood cholesterol before the bariatric surgery**? Yes/No
- Were you diagnosed with Type 2 diabetes before the bariatric surgery**? Yes/No

The following questions are only for women in childbearing age:

- Since the bariatric surgery have you become pregnant? Yes/No

  If yes- Was the pregnancy planned? Yes/no
- Have you been advised to delay pregnancy after surgery?

  Yes/No/Non-relevant
- If you received advice to delay pregnancy, what was the timeframe you were given?
  - Up to 6 months
  - 6 – 12 months
  - 12- 18 months
  - 18-24 months
  - Other (please state):__________

- Before surgery, were you advised about appropriate contraceptions to use after surgery?

  Yes/Partially/No/Non-relevant

  If Yes/Partially- by which practitioner?

  - Gynecologist/obstetrician
  - Bariatric surgeon
  - General practitioner
  - Registered dietitian
  - Psychologists or Social worker
  - Nurse
  - Social media
  - Other (please state):____________
- Have you used any contraceptive methods since the bariatric surgery?

  Yes/Partially/No/Non-relevant

  If Yes/Partially- What methods of the following?

  - Intrauterine device
  - Oral contraceptives
  - Birth Control Patch
  - Condoms
  - Other (Please state):_______________

- Has the method of contraceptive changed from the method you were using before surgery? Yes/No

- Please feel free to add any relevant information regarding these issues:
  ____
  ____

3. Hospitalization since the bariatric surgery

- Since the bariatric surgery, have you been admitted to a hospital? Yes/No

  If yes- What was the reason for the hospitalization?

  - Related to the bariatric surgery- please note the reason______
  - Not related to the bariatric surgery - please note the reason____________

4. Anthropometrics

- What is your height? ____(cm/feet/inches)
- What was your weight before the bariatric surgery? ____(kg/stones/pounds)
- What is your current weight? ____(kg/stones/pounds)
- What has been your lightest weight since the bariatric surgery? ____(kg/stones/pounds)

- How often did you weigh yourself in the last month?
    - More than once a day
    - Once a day
    - Few times per week
    - Once a week
    - Every couple of weeks
    - Once a month
    - Never

5. Gastrointestinal symptoms

This survey contains questions about how you have been feeling and what it has been like during the past month. Mark the choice that best applies to you and your situation.

- Have you been bothered by pain or discomfort in your upper abdomen or the pit of your stomach during the past month?

(1) No discomfort at all

(2) Minor discomfort

(3) Mild discomfort

(4) Moderate discomfort

(5) Moderately severe discomfort

(6) Severe discomfort

(7) Very severe discomfort

- Have you been bothered by heartburn during the past month? (By heartburn we mean an unpleasant stinging or burning sensation in the chest.)

(1) No discomfort at all

(2) Minor discomfort

(3) Mild discomfort

(4) Moderate discomfort

(5) Moderately severe discomfort

(6) Severe discomfort

(7) Very severe discomfort

- Have you been bothered by acid reflux during the past month? (By acid reflux we mean the sensation of regurgitating small quantities of acid or flow of sour or bitter fluid from the stomach up to the throat.)

(1) No discomfort at all

(2) Minor discomfort

(3) Mild discomfort

(4) Moderate discomfort

(5) Moderately severe discomfort

(6) Severe discomfort

(7) Very severe discomfort

- Have you been bothered by hunger pains in the stomach during the past month? (This hollow feeling in the stomach is associated with the need to eat between meals.)

(1) No discomfort at all

(2) Minor discomfort

(3) Mild discomfort

(4) Moderate discomfort

(5) Moderately severe discomfort

(6) Severe discomfort

(7) Very severe discomfort

- Have you been bothered by nausea during the past month? (By nausea we mean a feeling of wanting to throw up or vomit.)

(1) No discomfort at all

(2) Minor discomfort

(3) Mild discomfort

(4) Moderate discomfort

(5) Moderately severe discomfort

(6) Severe discomfort

(7) Very severe discomfort

- Have you been bothered by rumbling in your stomach during the past month? (Rumbling refers to vibrations or noise in the stomach.)

(1) No discomfort at all

(2) Minor discomfort

(3) Mild discomfort

(4) Moderate discomfort

(5) Moderately severe discomfort

(6) Severe discomfort

(7) Very severe discomfort

- Has your stomach felt bloated during the past month? (Feeling bloated refers to swelling often associated with a sensation of gas or air in the stomach.)

(1) No discomfort at all

(2) Minor discomfort

(3) Mild discomfort

(4) Moderate discomfort

(5) Moderately severe discomfort

(6) Severe discomfort

(7) Very severe discomfort

- Have you been bothered by burping during the past month? (Burping refers to bringing up air or gas from the stomach via the mouth, often associated with easing a bloated feeling.)

(1) No discomfort at all

(2) Minor discomfort

(3) Mild discomfort

(4) Moderate discomfort

(5) Moderately severe discomfort

(6) Severe discomfort

(7) Very severe discomfort

- Have you been bothered by passing gas or flatus (wind) during the past month?
  (Passing gas, wind or flatus refers to the need to release air or gas from the bowel, often associated with easing a bloated feeling.)

(1) No discomfort at all

(2) Minor discomfort

(3) Mild discomfort

(4) Moderate discomfort

(5) Moderately severe discomfort

(6) Severe discomfort

(7) Very severe discomfort

- Have you been bothered by constipation during the past month? (Constipation refers to a reduced ability to empty the bowels.)

(1) No discomfort at all

(2) Minor discomfort

(3) Mild discomfort

(4) Moderate discomfort

(5) Moderately severe discomfort

(6) Severe discomfort

(7) Very severe discomfort

- Have you been bothered by diarrhea during the past month? (Diarrhea refers to a too frequent emptying of the bowels.)

(1) No discomfort at all

(2) Minor discomfort

(3) Mild discomfort

(4) Moderate discomfort

(5) Moderately severe discomfort

(6) Severe discomfort

(7) Very severe discomfort

- Have you been bothered by loose stools during the past month? (If your stools (motions) have been alternately hard and loose, this question only refers to the extent you have been bothered by the stools being loose.)

(1) No discomfort at all

(2) Minor discomfort

(3) Mild discomfort

(4) Moderate discomfort

(5) Moderately severe discomfort

(6) Severe discomfort

(7) Very severe discomfort

- Have you been bothered by hard stools during the past month? (If your stools (motions) have been alternately hard and loose, this question only refers to the extent you have been bothered by the stools being hard.)

(1) No discomfort at all

(2) Minor discomfort

(3) Mild discomfort

(4) Moderate discomfort

(5) Moderately severe discomfort

(6) Severe discomfort

(7) Very severe discomfort

- Have you been bothered by an urgent need to have a bowel movement during the past month? (This urgent need to go to the toilet is often associated with a feeling that you are not in full control.)

(1) No discomfort at all

(2) Minor discomfort

(3) Mild discomfort

(4) Moderate discomfort

(5) Moderately severe discomfort

(6) Severe discomfort

(7) Very severe discomfort

- When going to the toilet during the past month, have you had the sensation of not completely emptying the bowels? (This feeling of incomplete emptying means that you still feel a need to pass more stool despite having exerted yourself to do so.)

(1) No discomfort at all

(2) Minor discomfort

(3) Mild discomfort

(4) Moderate discomfort

(5) Moderately severe discomfort

(6) Severe discomfort

(7) Very severe discomfort

Dyspepsia or indigestion is a group of symptoms that cause discomfort in the abdomen.

Mark the choice that best applies the severity of your dyspepsia symptoms in the last month

(0) None: no symptoms

(1) Mild: awareness of signs or symptoms, but easily tolerated

(2) Moderate: discomfort sufficient to cause interference with normal activities

(3) Severe: incapacitating with inability to perform normal activities

6. Defecations/bowel movements – frequency and quality

- What was the frequency of your defecations (bowel movements) in the last month?
  - 3 times a week or less
  - Once in 1-2 days
  - 1-2 times a day
  - 2-3 times a day
  - More than 3 times a day
- Please choose the option that best describes your defecation texture in the last month:
  Type ___

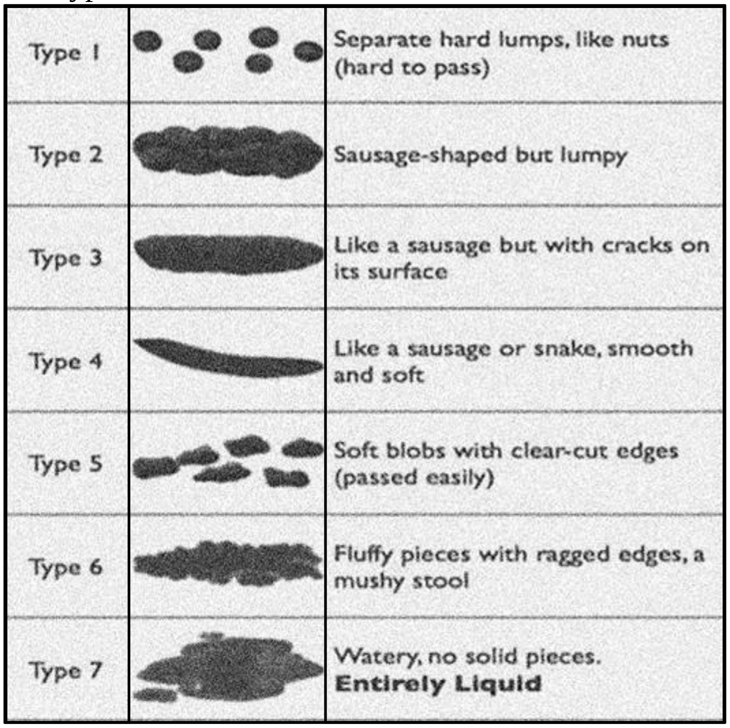

Type 1		Separate hard lumps, like nuts (hard to pass)
Type 2		Sausage-shaped but lumpy
Type 3		Like a sausage but with cracks on its surface
Type 4		Like a sausage or snake, smooth and soft
Type 5		Soft blobs with clear-cut edges (passed easily)
Type 6		Fluffy pieces with ragged edges, a mushy stool
Type 7		Watery, no solid pieces. **Entirely Liquid**

7. Information of Gastrointestinal symptoms

- Please fill the table below regarding taking prescription or over-the-counter medications/supplements for gastrointestinal symptoms since the bariatric surgery:

Symptoms	Did you take any medications/supplements for this symptom?	If Yes- note the product
**Heartburn**	Yes/No	
**Nausea**	Yes/No	
**Abdominal pain**	Yes/No	
**Bloating**	Yes/No	
**Flatulence (wind)**	Yes/No	
**Diarrhea**	Yes/No	
**Loose stools**	Yes/No	
**Constipation**	Yes/No	
**Other (Please state):______**	Yes/No	

- Did you receive an explanation on the expected gastrointestinal symptoms of the surgery in advance? Yes/Partially/No

  If Yes/Partially- by which practitioner?

  - Registered dietitian
  - Phycologists or Social worker
  - Bariatric surgeon
  - General practitioner
  - Nurse
  - Social media
  - Other (Please state):________________________________

8. Food tolerance after the bariatric surgery

- How would you rate the overall satisfaction regarding how you can eat presently?
  - Excellent
  - Good
  - acceptable
  - poor
  - very poor
- Why? (please feel free to comment) _____
- How many meals do you eat a day on average (including snacks)?___

- Among the following meals which one do you have? (can mark more than one)
  - ☐ Breakfast
  - ☐ Lunch (mid-day meal)
  - ☐ Dinner
- Which of these meals constitutes your daily main meal?
  - o Breakfast
  - o Lunch (mid-day meal)
  - o Dinner
- Do you eat between meals? Yes/No

  If Yes- How often?

  - o more than 3 times a day
  - o 2-3 time a day
  - o once a day
  - o more than 3 times a week, but less than once a day
  - o 2-3 times a week
  - o once a week
  - o less than once a week

  If yes, when? (can mark more than one)

  - ☐ Morning
  - ☐ Afternoon
  - ☐ Evening
- Can you eat everything? Yes/No
- More specifically, how can you eat each of the following food:

	**Easily**	**With some difficulties**	**Not at all**
**Red meat**	○	○	○
**White meat**	○	○	○
**Salad**	○	○	○
**Vegetables**	○	○	○
**Bread**	○	○	○
**Rice**	○	○	○
**Pasta**	○	○	○
**Fish**	○	○	○
**Pulses (such as lentils, chickpeas, peas, beans)**	○	○	○

- Are there other types of food that you cannot eat at all? (please state):______________

- Do you have any of the following food restrictions? (You can mark more than one choice)
  - ☐ Vegan (A plant based diet with no animal products)
  - ☐ Lacto-Ovo Vegetarians (Do not eat meat or fish, but eat dairy products and eggs)
  - ☐ Pescetarians (Do not eat meat, but eat fish, dairy products and eggs)
  - ☐ Abstain specific food due to food allergy (e.g. nuts, milk, soy)
  - ☐ Gluten free
  - ☐ Other (Please state):________
  - ☐ I have no food restriction
- Are there any socio-cultural aspects of that you need to take into consideration which may impact on eating, for example food choices, preparation and timing of meals? Yes/No
- Please feel free to add any relevant information regarding these issues:
  ____
  ____
- Do you vomit/regurgitate when you eat?
  - o Daily
  - o Often (more than 2 times a week)
  - o Rarely (less than 2 times a week)
  - o Never

9. Taste, smell and food aversion after the bariatric surgery

- Have you noticed any change in your appetite since your bariatric surgery? Yes/No
  If yes, please can you describe these changes:_________________________________
- Have you noticed any change in the taste of food or drink since your bariatric surgery? Yes/No
  If yes, please can you describe these changes:_________________________________
- Have you noticed any change in your sense of smell since your bariatric surgery? Yes/No
  If yes, please can you describe these changes:_________________________________
- Are there any foods or drinks that are repulsive or intolerable to you since your bariatric surgery? Yes/No
  If yes- please state what foods/drinks:_____________________________________
- Are there any foods or drinks that taste different to you since your bariatric surgery? Yes/No
  If yes- please state what foods/drinks:_____________________________________

- Are there any foods or drinks that smell different to you since your bariatric surgery? Yes/No
  If yes- please state what foods/drinks:___________________________________
- Do you feel that you eat less food because it does not taste or smell good? Yes/No
- Do you feel that you eat less food because you are simply less hungry? Yes/No
- How important is taste to your enjoyment of food? Important/Not important
- How important is smell to your enjoyment of food? Important/Not important

10. Quality of life and state of health

- Please rate your overall quality of life from 0-100 with 100 reflecting the 'best imaginable quality of life'.:______
- Please rate your overall state of health from 0-100 with 100 reflecting the 'best imaginable state of health':_______
- Please feel free to add any relevant information regarding these issues:
  ____
  ____

11. Adherence to postoperative eating recommendations

- Do you keep the following recommended behaviors for the <u>last month</u>
  (0- Not done, 1- Partially done, 2- Always done):

-Separation of fluids from solids	0/1/2
-Avoiding carbonated beverages (fizzy drinks)	0/1/2
-Drinking at least 8 cups/day of fluids (cup=200 ml)	0/1/2
-Chewing the food well in a relaxed manner	0/1/2
-Dividing food intake into 4–6 meals throughout the day	0/1/2
-Ending meals when feeling "comfortably full"	0/1/2
-Preferring to eat proteins (e.g. meat, chicken, fish, egg, dairies, soya, lentils) in most meals	0/1/2
- Preferring to eat solid foods (e.g. boiled egg, chicken breast, salad) over soft/crunchy/crispy foods (e.g. ice-cream, cookies, cakes, crackers) in most meals	0/1/2

12. Adherence to postoperative health recommendations

- During the last month, what kind of vitamins/minerals do you take and how often?

	Not taking	Daily (1 or more times per day)	Weekly (1-6 times per week)	Monthly/rarely (0-3 times per month)
**Multivitamin and minerals**	○	○	○	○
**Iron**	○	○	○	○
**Calcium**	○	○	○	○
**Folic acid**	○	○	○	○
**Vitamin B12 (IM injection)**	○	○	○	○
**Vitamin B12 (Orally)**	○	○	○	○
**Vitamin D**	○	○	○	○
**Probiotics**	○	○	○	○
**Other (Please state):______**	○	○	○	○

- During the last month, how many minutes do you invest in physical activity per week?
  - 0-30
  - 30-60
  - 60-90
  - 90-120
  - 120-150
  - >150
- During the last month, how many alcohol doses/units* do you drink per week?

*One Alcohol dose/unit (12-14 gr) is equal to 330 ml regular beer can (5% alcohol), 150 ml wine glass (12% alcohol) or 45 ml of other alcoholic drink (40% alcohol)

  - 0
  - 1-2
  - 3
  - >3 doses
- During the last month, have you experienced unusual hair loss? Yes/No

13. Attendance to the follow-up regime

- How many follow-up meetings did you have with each practitioner since the bariatric surgery?

	0	1-2	3-4	5-6	6-7	8-9	10 and above
**Registered dietitian**	○	○	○	○	○	○	○
**Psychologists/Social worker**	○	○	○	○	○	○	○
**Bariatric surgeon**	○	○	○	○	○	○	○
**Nurse**	○	○	○	○	○	○	○
**Pharmacist**	○	○	○	○	○	○	○
**Other (Please state):______**	○	○	○	○	○	○	○

- In your opinion, what is the importance of those follow-up meetings in a scale of 0 to 10? (10 represent very high importance and 0 not importance at all)

**Practitioner**	**Importance of follow up meetings**
**Registered dietitian**	
**Psychologists/Social worker**	
**Bariatric surgeon**	
**Nurse**	
**Pharmacist**	
**Other (Please state):_______**	

- Have you attended support group meetings since the bariatric surgery? Yes/No

If yes, how many times since the bariatric surgery? ______________________

- Please feel free to add any relevant information regarding these issues:

  ____

  ____

14. The use of social media for collecting professional data on the surgery

- Do you take part in local/national bariatric surgery groups through social media? Yes/No
- If you answer Yes - What is the reason for using social media?
  - Availability of 24/7
  - Peer support and interaction with other patients who underwent the same procedure
  - Don’t trust the healthcare professionals
  - Save time or money
  - Other (please state):____

15. Satisfaction from the bariatric surgery

- Check the statement below that best describes your opinion about your bariatric surgery:
  - ☐ I am glad that I had this procedure
  - ☐ I wish that I had chosen a different bariatric procedure
  - ☐ I wish that I had not undergone any bariatric procedure
